# Further Investigations and Parametric Analysis of Microstructural Alterations under Rolling Contact Fatigue

**DOI:** 10.3390/ma15228072

**Published:** 2022-11-15

**Authors:** Muhammad Usman Abdullah, Zulfiqar Ahmad Khan

**Affiliations:** NanoCorr, Energy & Modelling (NCEM) Research Group, Department of Design & Engineering, Bournemouth University, Poole BH12 5BB, UK

**Keywords:** bearing steel, diffusion, microstructural alterations, rolling contact fatigue, tempering

## Abstract

Bearing elements under rolling contact fatigue (RCF) exhibit microstructural features, known as white etching bands (WEBs) and dark etching regions (DERs). The formation mechanism of these microstructural features has been questionable and therefore warranted this study to gain further understanding. Current research describes mechanistic investigations of standard AISI 52100 bearing steel balls subjected to RCF testing under tempering conditions. Subsurface analyses of RCF-tested samples at tempering conditions have indicated that the microstructural alterations are progressed with subsurface yielding and primarily dominated by thermal tempering. Furthermore, bearing balls are subjected to static load tests in order to evaluate the effect of lattice deformation. It is suggested from the comparative analyses that a complete rolling sequence with non-proportional stress history is essential for the initiation and progression of WEBs, supported by the combination of carbon flux, assisted by dislocation and thermally activated carbon diffusion. These novel findings will lead to developing a contemporary and new-fangled prognostic model applied to microstructural alterations.

## 1. Introduction

Automotive components subjected to severe rolling cyclic loadings not only result in deterioration of interacting surfaces [1,2] but also cause subsurface microstructural damage [3,4]. This subsurface damage is pronounced in bearing steel under rolling contact fatigue (RCF) where the parent microstructure is evolved resulting in formation of microstructural alterations. The subsurface microstructural alterations can be observed under the optical microscope after Nital etching as a ring profile or semi-elliptical shape, depending on the plane of cut. The primary features involved in the altered microstructure are white etching bands (WEBs) preceded by dark etching regions (DERs) [4,5]. It has been reported that the microstructural features are formed due to the decay of parent martensite where carbon migrates within the matrix to form carbon-rich and carbon-depleted areas [6,7]. Extensive numerical and experimental research is available in the literature on bearing steel evolution and microstructural features; however, a comprehensive understanding is still needed to comprehend the general (physical and chemical) formation mechanism of DERs/WEBs.

DERs are considered to be an over-tempered form of martensite constituting a mixture of decayed microstructure, ferrite, and remaining martensite [5]. DERs are governed by the subsurface stress fields and their structure consists of deformed and misoriented martensite plates including reprecipitation of nano carbides, resulting in over-tempering of the microstructure [8]. WEBs are reported as disc-shaped ferrite plates surrounded by carbon-rich areas (also known as lenticular carbides, LCs) [9]. The mechanism by which DERs/WEBs are formed has been debated for the last few decades and redistribution of carbon within the white bands has been reported in the literature [7]. Earlier research [10,11] has indicated that WEBs are caused by stress and are developed as a result of subsurface plastic deformation. The structural changes observed during RCF have been attributed to recrystallisation, in which carbon atoms diffuse from supersaturated ferrite regions to create LCs. Carbon diffuses from ferrite interstitially to LCs and requires instigation energy to break their binding energies. This instigation energy has been reported as dissipated plastic energy during cyclic loadings [12,13,14]. Another theory [15] proposed that the carbon depletion within ferrite bands is caused by dislocation annihilation. Recently, Fu et al. [16] proposed a carbon migration theory based on dislocation gliding which indicated that carbon diffuses from ferrite bands to thicken neighbouring Lenticular Carbides. However, a bulk of the research has been centred on the migration of carbon within the ferrite matrix and could be restrained by either thermally driven diffusion or gliding dislocations [17]. A clear understanding of band formation is still required to resolve the ambiguity regarding white band formation.

Current research presents a systematic approach to understanding the development and formation mechanism of DERs/WEBs during RCF. Standard martensitic AISI 52100 bearings balls are tempered in a muffle furnace. The tempered and untempered ball samples are then subjected to specific RCF conditions in a rotary tribometer. Additionally, the RCF tests are performed and then subjected to similar tempering conditions. It is observed that the bearing pre-tempered RCF-tested ball samples showed significant microstructural alterations under the contact track as compared with untempered RCF-tested samples. The initially conducted RCF test exhibited the formation of DERs accompanying WEBs. After tempering, little to no difference in the WEB thickening is observed, however, randomly distributed white patch formation in the vicinity of DERs can be seen. Besides tempering, the static loading analysis is conducted to understand the effect of non-proportional loading histories. The bearing ball samples are subjected to the static load RCF test to evaluate the effect of lattice widening at extreme loading conditions. The mechanistic approach employed in current research is highly significant in evaluating RCF-induced subsurface alterations and can be employed for improving efficiency of analytical modelling applied in DER and WEB formation.

## 2. Experimental Setup

**Figure 1:**
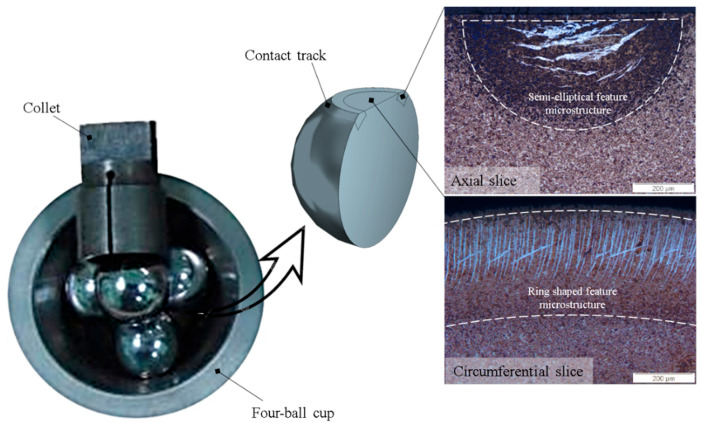
Rolling contact fatigue testing in a rotary tribometer.

RCF tests were conducted with the help of a four-ball configuration in a high-speed PLINT rotary tribometer, where three lower balls were kept (free to roll) in a cup which worked like an outer race of a bearing. In a four-ball test configuration, the upper ball (test sample) rotates on top of lower three balls and experiences 2.25 stress cycles [18]. A synthetic industrial ISO-Vg-320 oil was used in bath lubrication in order to avoid asperities interaction. Standard martensitic AISI 52100 (also known as 100Cr6) bearing balls of 12.7 mm diameter were used as test samples. Once the test was suspended, the upper ball in the collet was demounted, conditioned in an ultrasonic conditioning ultrasonic bath, and then sectioned into extremely thin slices in both axial and circumferential directions. A cubic boron nitride (CBN) cutter in a precision sectioning machine was employed with extensive cooling water to avoid the machining effects on the sample surface. After machining, the samples were cleaned, preserved via cold mounting, and polished with a 1 µm diamond suspension. Figure 1 demonstrates the sample preparation and resulting micrographs after Nital etching. Further details on the RCF experimentation, four-ball test setup, and respective sample preparation can be found in the authors’ previous research [18,19]. The acquired subsurface micrographs reveal the microstructure features as a semi-elliptical or as a ring-shaped geometrical entity via bright field microscopy after etching with Nital solution (3% nitric acid in ethanol).

## 3. Tempering Analysis

**Figure 2:**
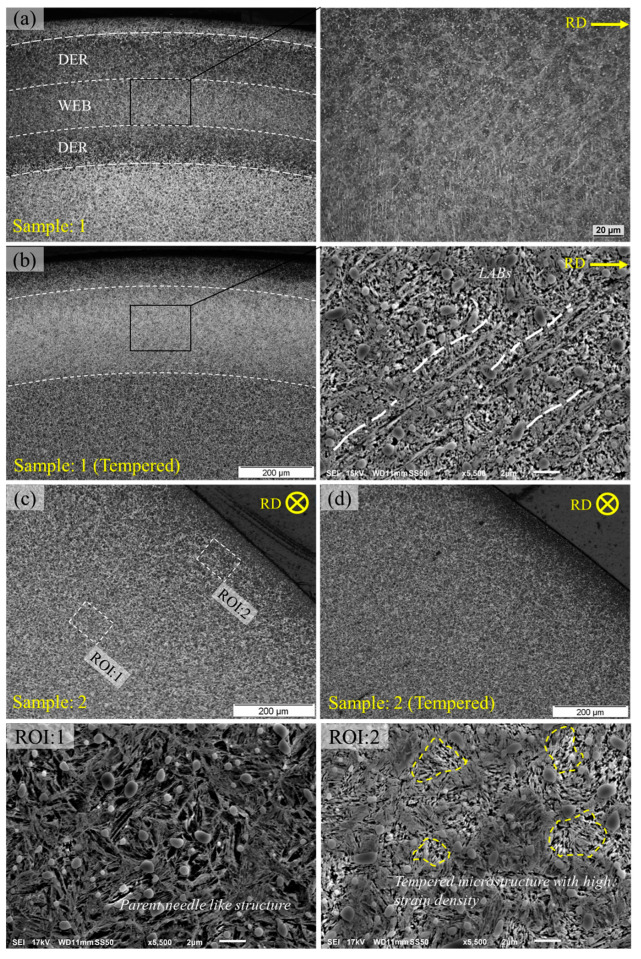
(**a**) Subsurface micrographs obtained at 6 GPa, 100 °C, after 37 million rolling cycles. (**b**) Tempering of RCF-tested sample shown in (**a**). (**c**) Micrographs obtained at 6 GPa, 100 °C, after 0.02 × 10^6^ rolling cycles. (**d**) Tempering of RCF-tested sample shown in (**c**). ROI:1 and ROI:2 represent regions of interest for parent structure and RCF damaged area respectively.

Standard AISI 52100 bearing ball samples were tested at 6 GPa peak contact pressure with 100 °C operational temperature, and 4051 rpm speed. Previous research reported [18] that the formation of microstructure features, especially the Lower Angle Bands (LABs), starts to form after 37 million cycles at 6 GPa and 100 °C. Therefore, it can be suggested that the initiating point for white bands formation exists near 37 million rolling cycles. The initial plastic shakedown of bearing elements during rolling cyclic fatigue is believed to occur in the very early stage of RCF, usually about 0.1% of total bearing life cycle [20]. Therefore, the bearing ball samples were RCF tested till 37 million and 0.02 million rolling cycles. The full details of conducted tests for tempering analysis are mentioned in Table 1. Figure 2 shows the optical micrographs of RCF-tested samples in axial and circumferential orientations. It can be seen that the bearing sample exhibits formation of LABs, nearly 10–20 μm in length, along with the formation of DER as observed in the circumferential orientation; refer to Figure 2a.

To understand the effects of thermal diffusion, the RCF-tested samples (1 and 2) were tempered in a muffle furnace at a temperature of 380 °C for 240 min. Standard martensitic bearing steel at such elevated temperatures is expected to undergo thermal tempering resulting in more carbon atoms available in the solute leading to enhanced formation of microstructural alterations. After tempering in a muffle furnace, the same sample was again polished and etched to remove any oxidation layer resulting from heat treatment. Figure 2b shows the formation of randomly distributed white patches in the vicinity of DERs. This white etching matter can be characterised as WEB. The inset of Figure 2a,b shows no difference in WEBs formation before and after tempering of bearing samples. The tempering temperature, as high as 380 °C resulted in the reprecipitation of the free carbon in the ferrite matrix which was segregated during the martensite–ferrite phase transformation. Additionally, the tempering of the RCF-tested sample also caused the overall reduction of DER which can only be seen in the upper and lower boundaries of white etching matter. However, no thickening of LABs or formation of higher angle bands (HABs) can be observed after heat treatment.

Figure 2c represents RCF investigated sample at 6 GPa maximum Hertzian contact pressure with 100 °C operational temperature till 0.02 million rolling cycles. In order to achieve such low stress cycles in a high-speed rotary tribometer, the spindle speed was reduced from 4051 rpm to 500 rpm. It should be noted that no microstructure features have been observed under such conditions as seen in the optical micrograph. It is difficult to justify the shakedown limit of certain bearing steel, however, it is speculated that under severe operating conditions, i.e., 6 GPa contact pressure, substantial plastic deformation will occur, which in turn will lead to a high dislocation density after several thousand rolling cycles. A similar sample was tempered to investigate the influence of carbon diffusion within areas of high dislocation density. It can be observed in Figure 2d that the heat treatment of RCF-tested samples does not influence the formation of DER which is a result of an over-tempered form of martensite, as suggested previously [21,22]. The regions of interest (ROI:1 and ROI:2) from Figure 2c are also displayed for parent martensitic structure and RCF-induced damaged area. It is evident from scanning electron micrographs that the original needle-like microstructure has been lost during the early shakedown stage of rolling fatigue cycles. The tempered martensite exists in the form of high dislocation regions due to subsurface plastic deformation.

The bearing ball samples were also tempered prior to RCF testing. For heat treatment, the ball samples were initially heat treated in a muffle furnace at 380 °C temperature for 240 min which resulted in a hardness drop of nearly 250 HV. The baseline hardness of the through-hardened bearing samples decreased from 782–810 HV (10 Kgf) to 525–560 HV (10 Kgf) after the heat treatment process. The tempered and untempered bearing ball samples were then subjected to rolling cyclic loading in the rotary tribometer discretely at 4 GPa and 5 GPa maximum contact pressure with 100 °C working temperature and a constant cyclic frequency of 4051 rpm.

**Figure 3:**
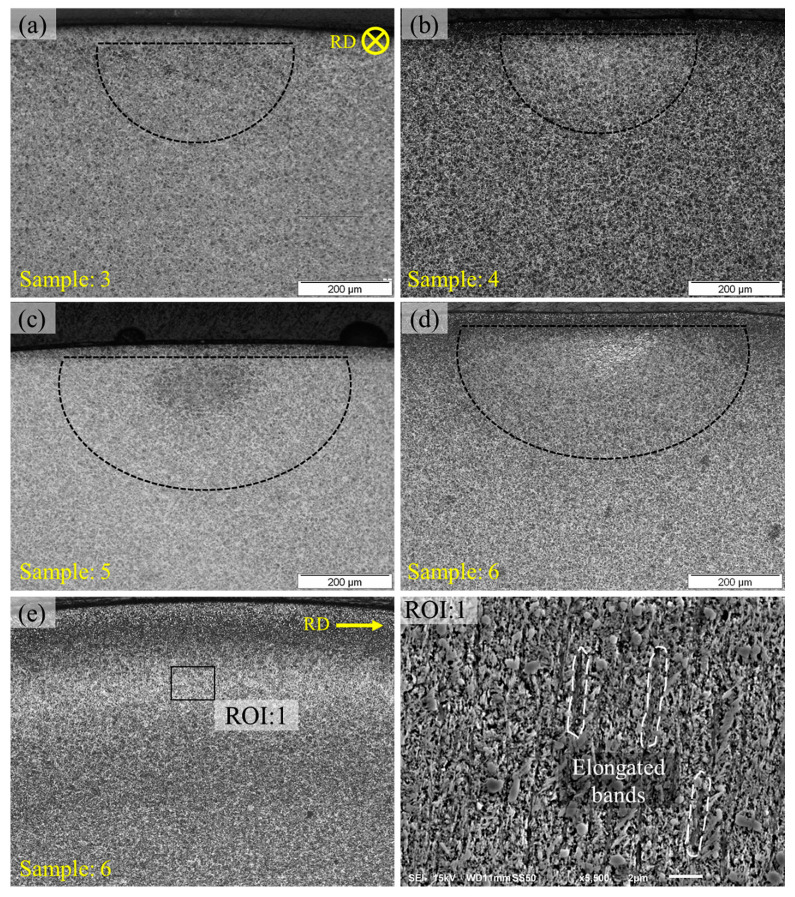
(**a**) Untempered and (**b**) tempered RCF-tested sample at 4 GPa, 100 °C, 4051 rpm. (**c**) Untempered and (**d**) tempered RCF-tested samples at 5 GPa, 100 °C, and 4051 rpm. The inset of circumferentially sliced sample (**e**) is shown in ROI:1.

It can be observed in Figure 3a,b that the bearing ball sample, after RCF testing at 4 GPa contact pressure, shows little to no formation of microstructural alterations, whereas the pre-tempered RCF test exhibits the development of microstructural features under the contact track. Correspondingly, the untempered bearing ball tested at 5 GPa contact pressure shows formation of DER in a very localised area under the centreline contact track, refer to Figure 3c, whereas the pre-tempered RCF-tested sample exhibits enhanced formation of white bands which can be observed in axial orientation as well as in circumferential orientation in Figure 3d,e. Scanning electron micrographs show these elongated band structures making steeper angles with the rolling direction (RD) and are described as premature HABs. The formation of premature HABs before LABs has been reported in the authors’ previous work [19] where stress as high as 6 GPa contact pressure has been reported to be a cause of WEBs sequence reversal. The development of HABs before LABs can be explained by considering white bands formation to be yield controlled rather than stress controlled as suggested by previous researchers [23]. It has been suggested that a threshold yield limit exists, beyond which the formation of white bands takes place in a preferable orientation, which is steeper to the RD.

The accelerated growth of white bands for pre-tempered RCF-tested samples suggests a significant effect of lowering of yield stress on microstructural alterations, resulting in subsequent subsurface plastic deformation. Moreover, the white bands are considered to be a softer structure as compared to the parent matrix [9], which can help promote accumulation of plastic strains. The change in yield stress and evolution of localised flow curves during the material phase transformation is presented in Section 6.

## 4. Effect of Thermal Diffusion

**Figure 4:**
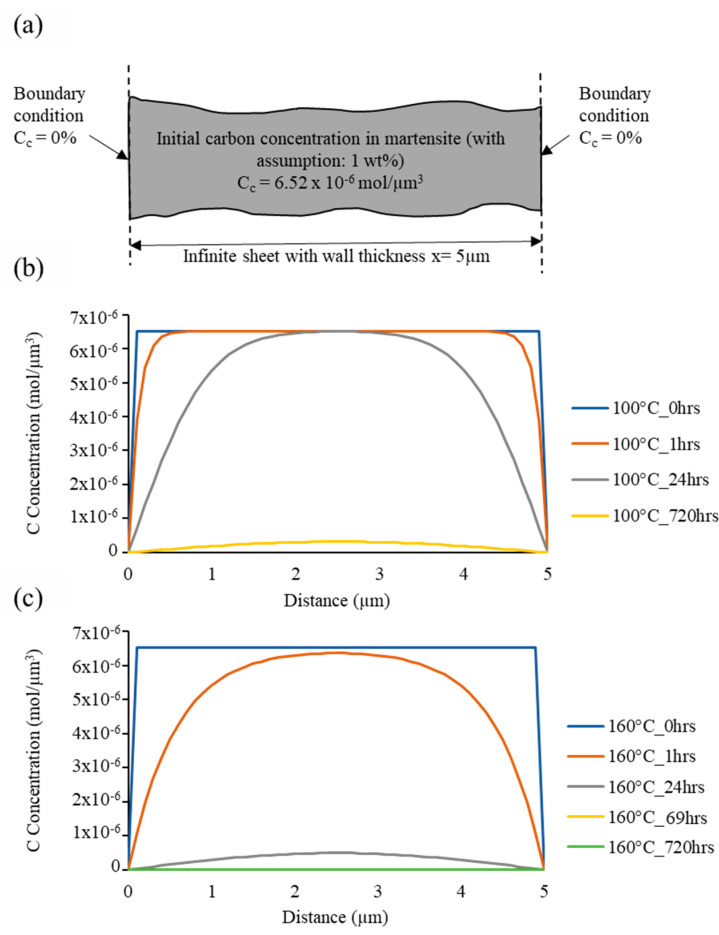
(**a**) Schematic for carbon diffusion in a 5 μm thick ferrite plate. Change in carbon concentration due to diffusion at (**b**) 100 °C and (**c**) 160 °C.

Figure 4a represents a schematic for carbon diffusion in a 5 µm thick ferrite plate. The initial carbon concentration of the matrix is assumed to be 6.52 × 10^−6^ mol/um^3^ as shown and the boundaries of the plate are assumed to be carbon-free, i.e., 0% carbon concentration. Equation (1) represents the generalised form of carbon flux J expressed as [24]

(1) $$J=-sD=\left[\frac{\partial \varphi }{\partial x}+k_{s}\frac{\partial }{\partial x}\left(\ln(\theta -\theta^{z}\right))+k_{p}\frac{\partial p}{\partial x}\right]$$

where D and s represent diffusivity and solubility, θ and p represent temperature and stress, where $k_{s}$ and k_p_ denote the temperature-driven and pressure-driven carbon diffusion factors, respectively. Assuming isotropic behaviour of carbon diffusion in alpha ferrite and neglecting the temperature and stress gradients in Equation (1), the change in carbon concentration of ferrite bands can be obtained relative to time. The diffusivity of carbon in α ferrite is defined in Equation (2) [25], where T represents temperature in kelvin.

(2) $$\log D=-0.9064-0.5199\left(\frac{10^{4}}{T}\right)+1.61\times 10^{-3}{\left(\frac{10^{4}}{T}\right)}^{2}$$

Figure 4b,c represent the estimated diffusion of carbon at 100° and 160 °C respectively. Complete depletion of a 5µm thick ferrite band is obtained at 160 °C temperature just after 69 h. It can be noted that the initial formation of WEBs (LABs/HABs) is observed after ~69 h of RCF testing under given conditions. The enhanced formation of HABs at 160 °C operating temperature is an indication of high carbon flux due to thermally activated carbon along with a large number of mobile dislocations. On the other hand, at 100 °C operating temperature, it takes more than 720 h (1 month) to diffuse out carbon in a 5 µm thick ferrite plate. It can be understood that at such slow rates of carbon diffusion, the transportation of carbon by dislocation glide becomes decisive. However, a typical run time for bearing components during service can reach tens of thousands of hours, where thermal diffusion of carbon should be accounted for in the formation of WEBs.

## 5. Static Load Analysis

To refute the migration of carbon as a result of dislocation gliding and understanding the carbon diffusion process during cyclic loading, static load analyses were carried out. The static load analysis follows a sequence where bearing balls are initially subjected to rolling cyclic loadings in a rotary tribometer followed by a static loading condition. During static loading, the upper ball in contact with the lower three balls was held static with the driver motor turned off. The contact track formed on the upper ball during rolling cyclic loading was marked precisely at the points of interactions. The time required to load the sample under static loading conditions was estimated from the numerical analysis discussed in Section 4. After the RCF test, the bearing ball sample was serially sectioned to investigate the statically loaded and offloaded subsurface regions. The testing parameters employed in the static load analysis are presented in Table 2.

**Figure 5:**
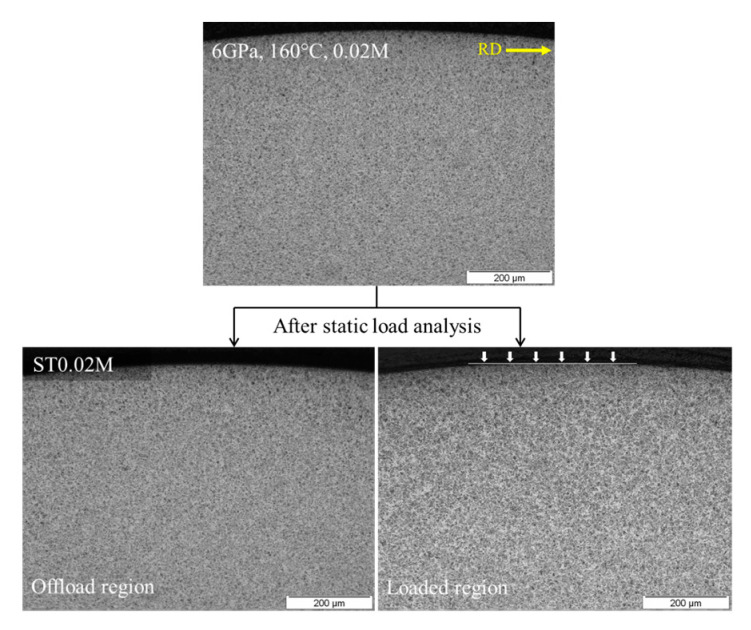
Circumferentially sliced micrograph acquired at 6 GPa, 160 °C after 0.02M rolling cycles. The static loaded and offload regions are also shown for ST0.02M.

Figure 5 represents the optical micrographs obtained at 6 GPa maximum contact pressure with 160 °C operational temperature. It was suggested previously [18] that DERs start to form at extreme testing conditions well before one million cycles. However, no feature microstructure can be observed till 20,000 rolling cycles. Having said that, at such extreme testing conditions, the bearing material undergoes subsurface plastic deformation with high dislocation density, as demonstrated previously in Figure 2. The RCF-tested bearing ball sample was then subjected to static loading conditions for 69 h, as illustrated in Table 2. As suggested by numerical simulation of the carbon flux across a thin ferrite plate, diffusion of carbon is highly likely to occur at 160 °C temperature after 69 h. Despite the numerical prediction and high dislocation density, no feature microstructure can be observed in the loaded and offloaded regions of ST0.02M sample. It should be noted that the optical micrograph for RCF testing and subsequent static loading analysis is acquired from different bearing ball samples.

**Figure 6:**
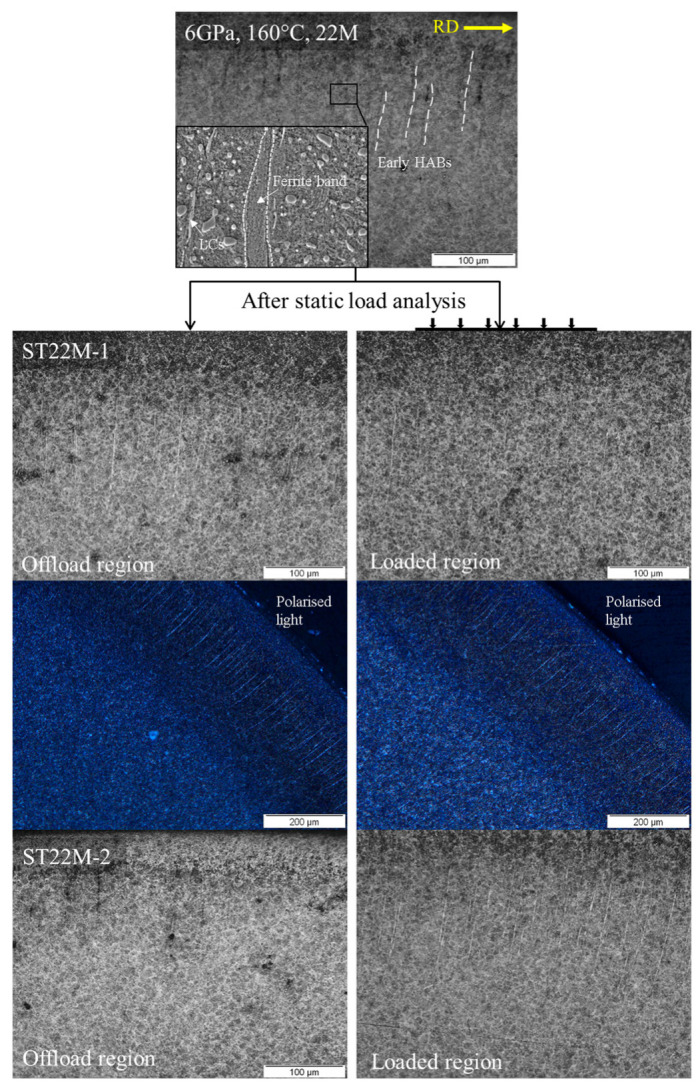
Circumferentially sliced micrographs obtained after 6 GPa, 160 °C, 22 M rolling cycles test, where the inset of figure shows microstructure of an early HAB. The loaded and offload regions of ST22M-1 and ST22M-2 are also shown after static load analysis. The high-contrast images are acquired from cross-polarised light.

Correspondingly, the optical micrographs obtained for 6 GPa, 160 °C, and 22 million rolling cycles are represented in Figure 6 which shows the formation of early HABs in the rolling direction. These initially developed HABs span nearly 100 μm in length and their microstructure contains deformed shear bands as shown in the inset image. These shear bands are formed due to the shear localisation and plastic deformation resulting in the martensite–ferrite phase transformation. As the maximum carbon solubility in the ferrite phase is nearly 0.02% [24], it is considered that the free carbon from such microstructural phase transformation can segregate at the boundaries and form carbon-rich plates termed as lenticular carbides (LCs). The transport of excess carbon atoms from the deformed ferrite matrix towards LCs can be accelerated with the help of carbon diffusion where thermal effects and lattice widening during static loading could lead to the thickening of pre-existing LCs. ST22M-1 and ST22M-2 represent the static loading analysis of RCF-tested samples till 69 h* and 100 h of testing time (* 69 h represents the testing time where initial WEBs can be observed under given loading conditions). It can be seen that despite the numerical prediction from Section 4, no thickening of LCs can be observed for either of the samples despite the prolonged tempering of the RCF-tested sample under static loading. Moreover, the static loaded regions do not show any notable difference in the growth of LCs as compared to the offload regions when observed in the high-contrast images from cross-polarised light microscopy.

**Figure 7:**
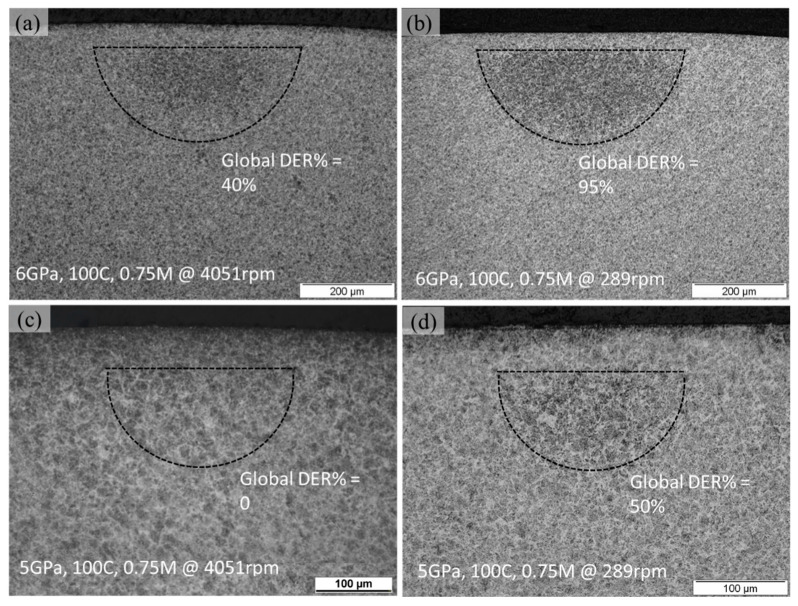
Axially sliced micrographs acquired at 6 and 5 GPa, 100 °C till 0.75 million cycles with (**a**,**c**) 4051 rpm (**b**,**d**) 289 rpm speed, respectively.

The additional RCF tests were carried out at 6 GPa, 100 °C till 0.75 million rolling cycles with variation in spindle speed, i.e., 4051 rpm and 289 rpm. Figure 7a,b representing the subsurface micrograph indicate that a higher cyclic speed results in decreased DERs formation. DERs formation can be quantified by a global DER%, characterising the fractional area of overall dark patches in the region of RCF-induced subsurface damage [19]. The global DER% calculated for slow cyclic speed gives an overall DER percentage of 95% which reduces to 40% at a higher cyclic speed. Likewise, at 5 GPa and 100 °C, a DER% of 50% can be observed at 289 rpm, whereas no DER can be seen at 4051 rpm speed. At low-stress frequency, more time is available for carbon to diffuse from ferrite to surrounding boundaries. In addition, the low-stress frequency promotes more carbon to be captured by dislocation during the gliding motion as suggested previously [8].

## 6. Discussions

**Figure 8:**
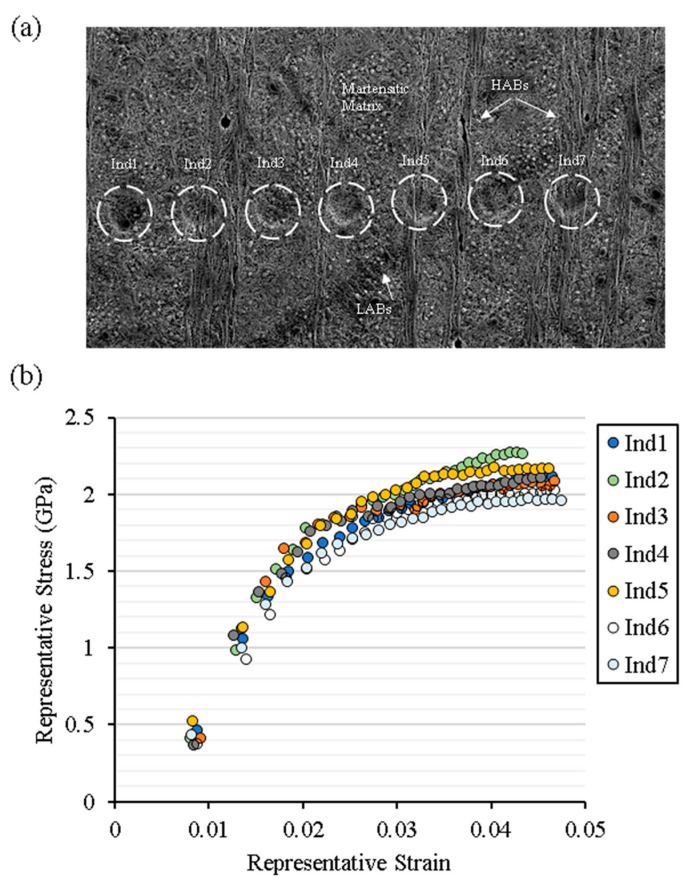
(**a**) Circumferentially sliced micrograph representing white etching bands (WEBs) and spherical nanoindentation performed. (**b**) Flow curves obtained from nanoindentation analysis of WEBs.

It is believed that the development of microstructure features involves degeneration of the parent structure and the microstructural phase transformation during plastic deformation. The subsurface plastic deformation takes place as a result of stress localisation and forms shear bands in the preferred planes due to non-proportional loading histories. Figure 8a represents the subsurface micrograph of feature microstructures containing HABs and LABs alongside the martensitic matrix. In order to evaluate the micromechanical properties of feature microstructure, a spherical conical indenter of 9 μm in diameter was employed. The loading was applied in a cyclic loading-partial unloading manner with a total penetration depth of 400 nm (assuming homogenous depth distribution). Each loading cycle yielded an elastic/plastic response of the target surface depending upon penetration depth. The semi-empirical approach to computing flow stress and representative strain from spherical indentation is described in Appendix A and further details are available in [26]. The localised flow curves are acquired from the indentation targets, shown in Figure 8a, and are plotted in Figure 8b). It is apparent from flow curves that bearing steel exhibits considerable strain hardening under cyclic loadings. The spherical indents Ind1, Ind3, and Ind4 demonstrate the martensitic structure’s stress–strain curve. Ind2 and Ind5 show LCs stress–strain curves, whereas Ind 6 and Ind7 depict stress–strain curves of LABs and HABs respectively. The martensitic matrix exhibits a baseline flow curve of bearing steel while significant hardening and softening can be observed in the regions of LCs and WEBs, respectively. A decrease in hardness in the carbon-depleted areas (ferritic grains) and an increase in hardness have been reported for carbon-rich areas (LCs) [9]. Moreover, a localised hardness map of WEBs has revealed hardness variation within a white band [19]. The variation in localised hardness arises due to grain size deviation, crystallography, change in chemical composition, and indenter penetration depth. The constitutive stress–strain curves obtained from spherical indentation suggest that the localised hardness can vary within a WEB, however, the global response of a WEB exhibit overall softening of the microstructure. In addition, the lowering of yield stress (softening), as suggested by the constitutive flow curves in the localised regions, indicates the ferritic nature of WEBs, which is the manifestation of RCF-induced accumulated plastic strain. The accumulation of plasticity within WEBs results in the formation of ferrite bands comprising dislocation cells.

The martensite–ferrite transformation and the formation of dislocation cells are an indication of high dislocation density in the areas of WEBs. With progressive loading cycles, the subsurface plasticity accumulates, and forms elongated ferrite bands, refer to Figure 6. These ferrite bands are oriented in the preferred slip system due to complex stress histories during rolling cyclic loading. The preferential planes of elongated ferrite bands have been described as the maximisation of normal relative stress across the contact track and have been explained in previous research work [19]. The carbon transport from ferrite to the grain edge becomes favourable. The free carbon in the ferrite region diffuses towards boundaries and precipitates as LCs. With continuous cyclic loadings, more and more carbon atoms become available in the ferrite matrix due to the dissolution of primary carbides and its transport to thicken the pre-existing carbide plates. To understand the transport of carbon, tempering and static load analysis have been conducted.

The heat treatment of bearing ball samples results in the reprecipitation of carbides. The post-tempering results also showed that the thermally activated carbon during thermal tempering cannot lead to formation of WEBs without external stress histories. It is noticeable that the pre-tempered RCF-tested sample showed the formation of white bands after 37 million rolling cycles along with DERs, whereas no white band is visible for the untempered RCF-tested sample. The poorly bonded carbides to the martensitic matrix during thermal tempering favour the formation of WEBs instead of DERs. The RCF test conducted with variation in stress frequency illustrated that a lower cyclic speed increases the time available for carbon to diffuse from ferrite to surrounding boundaries. Nevertheless, the low stress time also promotes more carbon to be captured by dislocation during the gliding motion. From Section 4, the numerical results of the carbon concentration gradient within supersaturated regions of ferrite predicted the diffusion of carbon from ferrite bands towards the surrounding matrix. However, the tempering results and static load analysis of bearing ball samples did not reveal the thickening of pre-existing LCs despite the prolonged tempering of RCF-tested samples under static loading. The static load analysis confirms that the WEBs development mechanism is determined by the non-proportional stress histories during cyclic loading which results in accumulated plastic strains to forming elongated ferrite bands.

It is evident from the comparative analyses that microstructural alterations are formed as a result of carbon flux, which is dislocation-assisted, in consort with thermally activated carbon diffusion. However, it is quite difficult to differentiate the individual effects of carbon diffusion and plastic deformation on the development of a feature microstructure. It is recommended to employ further material characterisation techniques to quantify the grain misorientations and lattice deformations within microstructural alterations. The mechanistic approach employed in the current study is highly significant in terms of evaluating the microstructural response of RCF-affected regions and can be used to improve the efficiency of analytical models for DERs/WEBs formation.

## 7. Conclusions

Bearing balls are tested systematically in a rotary tribometer for further evaluation of microstructural alterations. After RCF testing and subsequent parametric analyses, the following points are highlighted,

- The formation mechanism of WEBs accelerates at a temperature above the tempering condition of bearing steel. The excessive undissolved carbon arising due to thermal tempering is suggested to enhance the growth of WEBs rather than form DERs.
- The static load RCF test has validated that a complete rolling mechanism with a non-proportional stress history is essential for the development and unique orientation of WEBs.
- The localised hardness within WEBs can vary, however, the global response of white bands indicates softening of bearing material, as illustrated by localised flow curves.
- The development of a feature microstructure is a holistic effect of carbon diffusion and plasticity; and cannot be described solely with dislocation gliding or the carbon diffusion phenomenon, as presented previously in various research studies.

## Figures and Tables

**Table 1:** Experimentation conducted for tempering analysis.

Sample	Max Contact Pressure	Lubricant Temperature	Spindle Speed	Elapsed Cycles	Tempering Condition
1	6 GPa	100 °C	4051 rpm	37 million	380 °C for 240 min after RCF test
2	6 GPa	100 °C	500 rpm	0.02 million	380 °C for 240 min after RCF test
3	4 GPa	100 °C	4051 rpm	37 million	No heat treatment
4	4 GPa	100 °C	4051 rpm	37 million	380 °C for 240 min before RCF test
5	5 GPa	100 °C	4051 rpm	37 million	No heat treatment
6	5 GPa	100 °C	4051 rpm	37 million	380 °C for 240 min before RCF test

**Table 2:** Test conducted for static load analysis.

Test ID	Loading Condition	Peak Contact Pressure	Operating Temperature	Spindle Speed	Time for Static Load	Stress Cycles
ST0.02M	Rolling cyclic	6 GPa	160 °C	500 rpm	-	20,034
	Static			-	69 h	-
ST22M-1	Rolling cyclic			4051 rpm	-	22,001,035
	Static			-	69 h	-
ST22M-2	Rolling cyclic			4051 rpm	-	22,067,262
	Static			-	100 h	-

**Figure A1:**
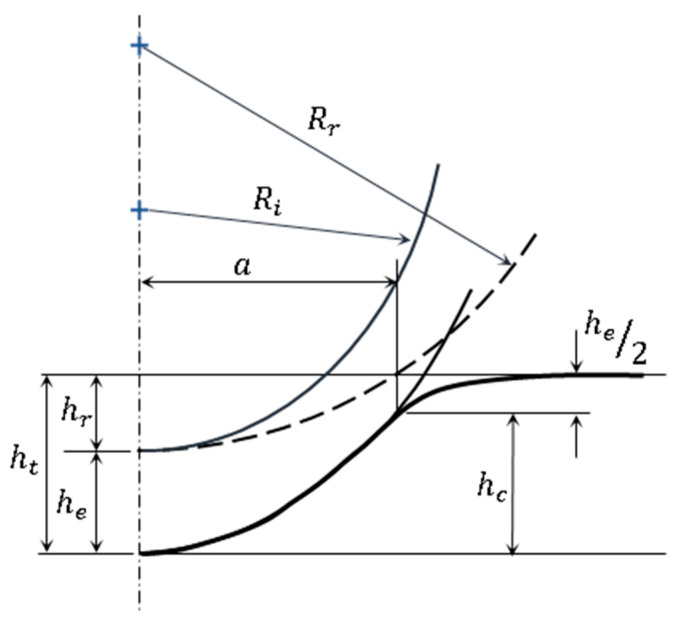
Schematic for spherical indentation.

## Data Availability

Not applicable.

## Appendix A

Nanoindentation analysis is performed at a single point with continuously increasing multiple cyclic loading and unloading. For this, a 9 µm spherical indenter is used with NanoTest VANTAGE Micromaterials. The contact area is determined by using the data from spherical indentation analysis and the given radius of diamond indenter, as demonstrated in Figure A1. Subsequently, hardness H, Young’s modulus E, stress Y, and strain are calculated. This cyclic loading–unloading procedure is expressed as the Field & Swain analysis [27]. The spherical indentation has been employed previously [28,29] for mechanical property evaluation of small volumes of materials.

With multiple loading cycles during spherical indentation, the total penetration depth $h_{t}$ (elastic + plastic component) depends on total applied load $P_{t}$. During partial unloading, the recovered depth $h_{s}$ depends upon the reduced load $P_{s}$. Once the entire load is removed, there will be an associated residual depth $h_{r}$ which can be expressed as [29],

(A1) $$h_{r}=\frac{r\cdot h_{s}-h_{t}}{r-1}$$

Whereas r is a factor described as,

(A2) r=PtPs23

The elastic depth ($h_{e}$) and plastic depth ($h_{p}$) during an individual loading cycle can be computed as,

(A3) $$h_{e}=h_{t}-h_{r}$$

(A4) $$h_{p}=\frac{h_{t}+h_{r}}{2}$$

Plastic depth and radius of spherical indenter R are used to calculate contact circle radius ‘a’ as given below, which is in the original surface plane at every indentation cycle.

(A5) $$A=\sqrt{2\;{R\;h}_{p}-h_{p}^{2}}$$

Hardness H, the yield/flow stress $Y_{r}$, and subsequent representative strain $\varepsilon_{r}$ can be computed for load step i=1ton as follow,

(A6) $$H_{i}=\frac{P_{t_{i}}}{A_{i}}=\frac{P_{t_{i}}}{{\pi a}_{i}^{2}}$$

(A7) $$Y_{r_{i}}=\frac{H_{i}}{3}$$

(A8) $$\varepsilon_{r_{i}}=0.2\frac{a_{i}}{R}$$

Equations (A1)–(A8) [27,29] can be used to extract micromechanical properties from the indented surface with the help of cyclic loading–unloadings using a conical spherical indenter.
